# An Evaluation of the Antibacterial Properties of Tormentic Acid Congener and Extracts From *Callistemon viminalis* on Selected ESKAPE Pathogens and Effects on Biofilm Formation

**DOI:** 10.1155/2020/8848606

**Published:** 2020-11-07

**Authors:** Tafadzwa Chipenzi, Genuine Baloyi, Tatenda Mudondo, Simbarashe Sithole, Godloves Fru Chi, Stanley Mukanganyama

**Affiliations:** ^1^School of Pharmacy, College of Health Sciences, University of Zimbabwe, Mt. Pleasant, Harare, Zimbabwe; ^2^Department of Biochemistry, University of Zimbabwe, Mt. Pleasant, Harare, Zimbabwe; ^3^University of YAOUNDE 1, P.O. Box 812, Yaoundé, Cameroon

## Abstract

ESKAPE pathogens, namely, *Enterococcus faecium*, *Staphylococcus aureus*, *Klebsiella pneumoniae*, *Acinetobacter baumannii*, *Pseudomonas aeruginosa,* and *Enterobacter* species, are responsible for a majority of all healthcare-acquired infections (HAI). The bacteria cause nosocomial infections in immunocompromised patients. Extracts from *Callistemon viminalis* have been shown to have antibacterial, antifungal, and anti-inflammatory activities. Tormentic acid congener, a pentacyclic triterpene saponin, was isolated from *C. viminalis* leaves. This study aimed to investigate the antibacterial effects of tormentic acid congener and leaf extracts on biofilm formation by *A. baumannii*, *S. aureus*, *S. pyogenes,* and *P. aeruginosa*. The antibacterial effects were determined by the microbroth dilution method, and ciprofloxacin was used as the standard antibacterial drug. Biofilm formation and detachment assays were performed using crystal violet staining. Production of extracellular polymeric DNA and polysaccharides from biofilms was also determined. Tormentic acid congener showed time-dependent antibacterial activity against *P. aeruginosa* with a MIC of 100 *µ*g/ml and caused significant protein leakage. Antibacterial activity was found when tormentic acid congener was tested against both *S. aureus* and *P. aeruginosa*. The MICs were found to be 25 *µ*g/ml and 12.5 *µ*g/ml for *P. aeruginosa* and *S. aureus* cells, respectively. *S. pyogenes* was found to be susceptible to tormentic acid congener and the hydroethanolic extract with an MIC of 100 *µ*g/ml and 25 *µ*g/ml, respectively. *A. baumannii* was found not to be susceptible to the compound or the extracts. The compound and the extracts caused a significant decrease in the biofilm extracellular polysaccharide content of *S. pyogenes*. The extracts and tormentic acid congener caused detachment of biofilms and decreased the release of extracellular DNA and capsular polysaccharides from biofilms of *P. aeruginosa* and *S. aureus*. Tormentic acid congener and extracts, thus, have significant antibacterial and antibiofilm activities on these selected ESKAPE bacteria and can act as source lead compounds for the development of antibacterial triterpenoids.

## 1. Introduction

ESKAPE pathogens are responsible for two-thirds of all healthcare-associated infections [1]. The Infectious Diseases Society of America (ISDA) formulated an acronym ESKAPE to emphasize the group of pathogens that cause hospital infections and effectively “escape” the effects of antibacterial drugs [2]. Gram-positive pathogens, vancomycin-resistant enterococci (VRE) and methicillin-resistant *Staphylococcus aureus* (MRSA), and Gram-negative pathogens, *Pseudomonas aeruginosa* and *Acinetobacter baumannii* as well as extended spectrum-lactamase producing (RSBL) or carbapenem-resistant Enterobacteriaceae (CRE) were used [2]. Approximately 10–15% of the nosocomial infections on a worldwide scale are caused by *P. aeruginosa* [3]. Nosocomial infections are responsible for hospital-acquired infections largely in immunocompromised patients [4].

*K. pneumoniae* and *P. aeruginosa* have been found to cause life-threatening hospital infections in critically ill individuals [5]. These two pathogens have acquired resistance against some of the common antibacterial drugs [6]. *K. pneumoniae* belongs to the family Enterobacteriaceae [7], and it is one of the most common pathogens associated with hospital-acquired infections [8]. *K. pneumoniae* naturally inhabits the gastrointestinal tract microbiome of healthy humans and animals [9]. *S. aureus* is a Gram-positive bacterium carried in the nostrils of approximately 30% of the people [10]. In most cases, *S. aureus* does not cause any harm; however, in hospitalised immunocompromised people, it may cause infections. These bacteraemia invasions affect people with underlying lung disease, including those on mechanical ventilators and endocarditis, and this can lead to heart failure or stroke [10]. *S. pyogenes* is a Gram-positive, nonmotile, nonspore forming, catalase-negative cocci that occur in pairs or chains [11]. *S. pyogenes* infections cause pharyngitis and are responsible for up to 33% of diagnosed cases of sore throat in children and up to 10% in adults [12]. ESKAPE pathogens have become a major cause of morbidity and mortality all over the world [13]. *A. baumannii* has the ability of surviving for long periods on hospital surfaces and equipment. This is aided by its ability to develop resistance to multiple antibiotics [14] leading to outbreaks in clinical settings [15].

Biofilm formation is a bacterial strategy to survive under adverse conditions [16]. Bacteria develop resistance to antimicrobial agents as well as thrive in seemingly severe conditions, and this has been attributed to their ability to form biofilms [17]. The production of extracellular polymeric substance (EPS) protects bacterial cells from environmental damage, which leads to these cells developing resistance to antibiotics [18]. There is a continued evolution of dangerous multidrug-resistant bacteria that has led to significant increases in morbidity and mortality due to bacterial infections. Many of the currently prescribed antibacterial drugs have significant adverse side effects [19]. There is urgency for the development of new highly effective and safe antibacterial agents from natural products specifically the complementary and alternative medicines (CAM). The use of different plant sources to search for antibacterial agents has been shown to be a promising approach [20].

Plants of the *Callistemon* genus are known to possess antifungal, antioxidant, antithrombin, anti-inflammatory, antidiabetic, antibacterial, and herbicidal activities [21]. *Callistemon viminalis*, also known as bottlebrush, is an ornamental plant which belongs to Myrtaceae family. Extracts from *C. viminalis* have been reported to have various medicinal properties including antibacterial, antifungal, and antioxidant activities [22]. Compounds in *C. viminalis* have shown antibacterial activity against *S. aureus* and *E. coli* with an inhibitory zone diameter about 16–20 mm [23]. Phytochemical analysis of *C. viminalis* leaves has demonstrated the presence of phenolic, triterpenoid, flavonoid, saponin, steroid, alkaloid, tannin, carbohydrate, amino acid, and protein compounds [24]. Terpenoids compounds in *C. viminalis* have shown antimicrobial properties for some bacteria [25]. Triterpenoids are chemical compounds from a class of terpenoids, with chemically characterised six isoprene units and a total of 30 carbon atoms [26]. Triterpenoids are widely distributed secondary metabolites found in many genera of plants and other living organisms, and they exist in different states [27]. Several triterpenes have been isolated from fungi and plant species for investigation into antibacterial agents against *P. aeruginosa* [26]. Several studies have found triterpenes to possess antibacterial effects on *K. pneumoniae* [26].

Tormentic acid can be isolated from several plants which include *C. viminalis*, *Sarcopoterium spinosum* [27], and *Potentilla chinensis* [28] among others. The main objective of the study was to evaluate the antibacterial activity of tormentic acid congener, a triterpenoid isolated from *C. viminalis*, on *K. pneumoniae*, *P. aeruginosa, S. aureus, S. pyogenes,* and *A. baumannii*. In addition, we investigated the effects of tormentic acid congener on biofilm production of *S. aureus*, *S. pyogenes*, *P. aeruginosa,* and *A. baumannii.*

## 2. Materials and Methods

### 2.1. Reagents

The chemicals and solvents used in this study were all obtained from Sigma-Aldrich Chemicals Company (Munich, Germany). Dichloromethane, ethanol, methanol, and water were used for extraction of *Callistemon viminalis* crude extracts. Dimethyl sulfoxide (DMSO) was used for dissolving the extracts, ciprofloxacin (the standard antibiotic drug), and tormentic acid congener, and 3-(4,5-dimethylthiazol-2-Yl)-2,5-diphenyltetrazolium bromide (MTT) was used as an indicator of cell viability after carrying out assays. The bacterial species *P. aeruginosa* (NCTC 10662), *S. pyogenes*, *K. pneumoniae* (ATC700603), *A. baumannii* (CECT(R) 911), and *S. aureus* (NCTC 6571) were obtained from Merck (Darmstadt, Germany). Before resuscitation, the cells were kept in a −80°C freezer as 1 ml stock strains in 50% glycerol. Luria agar base, Miller, was used to plate cells on agar plates, and all the cells were cultured in Luria broth base, Miller. The tormentic acid congener used was isolated from *Callistemon viminalis* and characterised by NMR and mass spectrometry. All assays were performed in the biological safety cabinet Bioflow-II Labotec, Model 650. The incubator shaker, Model number S1-300 (Jeiotech Co., Korea), and the incubator SI-300 were used for all incubations carried out. For analysis of cell viability using absorbance, a Tecan Genios Pro microplate reader (Tecan Group Ltd, Männedorf, Austria) was used to record the results from a 96-well microwell plate. GraphPad Prism6 (San Diego California, USA) was used to record and analyse the results. For centrifugation, the centrifuge, Rotafix-32 Hettich Zentrifugen Microcent 94–2 Eppendorf centrifuge 541(Sigma-Aldrich Co. Darmstadt, Germany) was used.

### 2.2. Plant Collection and Preparation

The leaves of *Callistemon viminalis* were collected in Harare, the University of Zimbabwe, 17.7840°S, 31.0530°E. The plants had been previously authenticated by a taxonomist from the National Herbarium and Botanic Gardens in Harare, Zimbabwe. The leaves of *C. viminalis* were separated from the branches and predried using a Labcon orbital incubator (Labotech Co., Cape Town, SA) at 60°C. The dried leaves were ground using a pestle and mortar to produce approximately 4 g of a powdered sample.

### 2.3. Preparation of Plant Extracts

The leaves were washed with distilled water then dried in an oven (Memmert, Model 400, D06060) at 60°C. The dried leaves were pounded in a traditional mortar and pestle and sieved to obtain a powder. Two solvents were prepared, one with 50% v/v DCM: MeOH and the other one with 50% v/v EtOH:H_2_O. The powdered leaves and solvents were mixed in the ration 1 : 10, respectively. The leaves were left soaked in solvents for 3 days after which filtration was performed to obtain the extract. Filtration was done twice, first using cotton wool and using a filter paper thereafter. After filtration, the excess solvent was evaporated using a Buchi RII rotavapor (BÜCHI Labortechnik AG, Postfach, Switzerland), and the crude extract was dried under a fan.

### 2.4. Column Chromatography on Silica Gel

The leaf extract was run on a column with 100% hexane initially as the eluting solvent. Batch gradient system column elution was employed starting with a solvent of low polarity to that with high polarity. The batch gradient system was a 20-step gradient elution with a gradual increase of polarity with 100% ethyl acetate. Methanol was then added to 100% EA up to 90% EA and 10% methanol. Fractions of 250 ml were collected and concentrated using a Buchi RII rotary evaporator (BÜCHI Labortechnik AG, Postfach, Switzerland). Thin-layer chromatography was used for the analysis of the collected fractions. The fractions with similar retardation factor values were pooled. The pooled fractions were left to evaporate to dryness for crystals to form. Single spots observed on the developed chromatograms under UV (354 and 365 nm) after staining with sulphuring acid were deemed pure and were subjected to NMR analyses.

### 2.5. NMR Analyses and Determination of the Mass of the Isolated Compound

^1^H NMR spectra were recorded at 400 MHz and ^13^C NMR spectra at 100 MHz. The chemical shifts for ^1^H NMR and ^13^C NMR were referenced to TMS via residual solvent signals (^1^H, CDCl_3_ at 7.26 ppm; ^13^C, CDCl3 at 77.36 ppm; ^1^H, DMSO*-d*_*6*_ at 2.45 ppm; ^13^C, DMSO*-d*_*6*_ at 39.43 ppm, ^1^H, CD_3_OD at 3.31 ppm; and ^13^C, CD_3_OD at 49.0 ppm). Two-dimensional (2D) NMR experiments were run using standard pulse sequences. Molecular formulae were determined by electrospray ionization with a 7T hybrid ion trap and a TOF detector running in a positive or negative mode. Tormentic acid congener was identified as a pure compound.

### 2.6. Antibacterial Susceptibility Tests

Bacteria were grown in Luria broth base, Miller (Sigma-Aldrich), supplemented by casein acid hydrolysate 10 g/L, yeast extract 5 g/L, and sodium chloride 5 g/L. The antibacterial effects of the compound tormentic acid congener as well as those for the DCM:MeOH and EtOH:H_2_O leaf extracts of *C. viminalis* were investigated by adapting the microbroth dilution procedure used by Vipra et al. [29]. Briefly, the tormentic acid congener as well as the DCM:MeOH and EtOH:H_2_O leaf extracts of *C. viminalis* were dissolved to give a final concentration of 1% DMSO and 100 *µ*g/ml. The resultant mixtures were serially diluted in a 2-fold manner with media containing 1% DMSO up to a minimum concentration of 12.5 *µ*g/ml. For all experiments, bacterial cells exponentially growing were standardised using 0.5 McFarland's standard solution to give a cell suspension with a concentration of 1 × 10^6^ CFU/ml.

Tormentic acid congener, extracts, cells, and Luria broth were diluted into the 96-well microplate wells (Greiner Bio-One, Sigma-Aldrich, St. Louis, MO, USA). Each well contained a final volume of 200 *µ*l. A positive control containing ciprofloxacin (highest concentration of 10 *µ*g/ml) was set up in each well-containing media and bacterial growth culture. Relevant sterility and negative controls were set up containing media only and media with extract, respectively. Preincubation and postincubation cell density measurements were determined. After postincubation measurements, visualisation of viable cell growth on the plate was investigated by adding 20 *µ*l of 3-(4, 5-dimethylthiazol-2)-2, 5-diphenyltetrazolium bromide (MTT) solution (1 mg/ml) to each well. The 96-well microplate was then covered and placed in an incubator for 2 hours.

### 2.7. Time-Kill Assays of Tormentic Acid Congener on *P. aeruginosa*

The time-kill assays for tormentic acid congener on *P. aeruginosa* were performed in LB culture medium after carrying out serial dilutions starting from 100 to 12.5 *µ*g/ml. After standardisation of cells using the 0.5 McFarland solution, ciprofloxacin was used as a control standard and untreated cells were used as a positive control. Aliquots of diluted cell suspension and tormentic were added to a microplate wells plate in equal volumes to produce a total volume of 200 *µ*l. The microplate was incubated, and then, the absorbance was measured under suitable conditions for varied time intervals (0, 8, 10, 24, 26, 28, 30, and 32 hours). The results of the absorbance representing cell densities were used to plot time-kill graphs for 100 *µ*g/ml, 50 *µ*g/ml, 25 *µ*g/ml, 12.5 *µ*g/ml, and the control. MTT was then added to the 96-well microplate to determine cell viability. The bactericidal effect was obtained from observing the graph with a lethality percentage of 90% for 6 hours, which is equivalent to 99.9% of lethality for 24 hours. This method was used to further confirm the MIC of tormentic acid congener on *P. aeruginosa*.

### 2.8. Effects of Combining Ciprofloxacin and Tormentic Acid Congener on *P. aeruginosa*

The checkerboard assay was used to determine the effects of combining ciprofloxacin and tormentic acid congener on *P. aeruginosa* in consideration of their individual effects against this bacterium. This assay was aimed at determining and observing the presence of antagonism, synergism, and zero interaction between tormentic acid congener and ciprofloxacin [30]. Since there was 88% agreement between time-kill and checkerboard assay, this method was used to confirm the effects of tormentic acid congener on *P. aeruginosa* as shown by time-kill assays [31] and to calculate the fractional inhibitory concentration. After standardisation of cells using the 0.5 McFarland scale, 100 *µ*l of the inoculum was added to each well in a sterile 96-well microplate. Equal volumes of 50 *µ*l tormentic acid congener and 50 *µ*l ciprofloxacin were added to the wells. The plate was incubated at 37°C, and absorbance of the cells was measured at varied time intervals (0, 8, 10, 24, 26, 28, 30, and32 hours). The results of the absorbance were used to plot time-kill graphs for 100 *µ*g/ml, 50 *µ*g/ml, 25 *µ*g/ml, 12.5 *µ*g/ml, and control. The fractional inhibitory index (FICI) was calculated with the concentrations in the first nonturbid well found in each row and column of the microplate after extrapolating line graphs [32]. The different combinations are categorised as synergistic if FICI  is ≤ 0.5, antagonistic when FICI  > 4, and indifferent if 0.5  <  FICI  ≤ 4 [32]. The FICI was calculated using the following equation [32]:

(1) $$\begin{array}{c} {\text{FICI}}_{A/B}=\frac{\text{MICA}\left(\text{combination}\right)}{\text{MICA}\left(\text{alone}\right)}+\frac{\text{MICB}\left(\text{combination}\right)}{\text{MICB}\left(\text{alone}\right)}. \end{array}$$

### 2.9. Effects of Tormentic Acid Congener on Protein Leakage

From precultured *P. aeruginosa* cell suspension, a volume of 200 *µ*l was subcultured in 200 ml of Luria broth base in a 1-litre container, and the cells were incubated overnight at 37°C at 100 rpm. The subcultured cells were centrifuged at 3500 rpm for 4 minutes, and the supernatant was removed. The pellet of *P. aeruginosa* cell suspension was diluted with 0.9% normal saline to produce absorbance of 1.5 OD using a centrifuge (Rotafix-32 Hettich Zentrifugen Microcent 94–2 Eppendorf centrifuge 541). To triplicates of labelled tubes A, B, C, D, and E, 6 ml of diluted cells was added. To tube A, 150 *µ*l of tormentic acid congener was added to make a final concentration of 100 *µ*g/ml. To tube B, 2.4 *µ*l of ciprofloxacin was added to make up a final concentration of 0.16 *µ*g/ml. To the control tube C, 1200 *µ*l of SDS was added, to tube D, 180 *µ*l of DMSO was added, and untreated cells were added to tube E. The tubes were centrifuged at 3500 rpm in the centrifuge for 4 minutes, and the pellets were discarded; the supernatants were used for protein determination assay. An exact volume of 150 *µ*l of the supernatants was separately added to a labelled sterile 96-well plate. A calibration curve of concentration 0–50 *µ*g/ml of the BSA stock was made. To each well, 150 *µ*l of Bradford's reagent was added, and the optical densities of the wells were read at 590 nm using a Tecan Genios Pro microplate reader (Tecan Group Ltd, Männedorf, Austria).

### 2.10. Effect of Extracts and Tormentic Acid Congener on Biofilm Formation

#### 2.10.1. Biofilm Formation Procedure

For this assay, precultured cells were then centrifuged at 3500 rpm for 15 minutes in a Rotafix-32 Hettich Zentrifugen. The supernatant of the centrifuged cell culture was discarded, and 20 ml of 0.5 M phosphate-buffered saline prepared from sodium dihydrogen phosphate as the acidic salt and disodium hydrogen phosphate as the basic salt at pH 7.20 was used to wash the cells. The cell suspension was centrifuged again at 3500 rpm for 15 minutes, the supernatant was discarded, and 5 ml of sterile media was added and hand shaken to the dissolve the cell pellet in the media. A 2 ml volume of standardised cells was added to each well of a 24-well plate (Sigma-Aldrich Co. Darmstadt, Germany). Thereafter, the cells were allowed to adhere to the wells by incubating the cells at 37°C for 2 hours. During the incubation period, stock concentrations of test solutions were prepared to give a final concentration of 100 *µ*g/ml and 10 *µ*g/ml for ciprofloxacin. After the incubation, 500 *µ*l of antibacterial test solutions was added to the wells and left for further incubation for 72 hours at 37°C in a humid nonshaking incubator. After incubation, the contents of each well were gently discarded, and the plate was gently washed three times with 0.5 M phosphate buffer saline at pH 7.20. After washing, the plate was inverted on a paper towel for 15 minutes to drain off excess liquid, and the biofilms were subsequently fixed by incubating the plate at 60°C for 1 hour in an oven. After incubation, the biofilms were quantified as follows: 2.5 ml of 0.1% crystal violet was added to each well, the plate was further incubated at room temperature for 20 minutes before the crystal violet was discarded, and the plate was gently washed with sterile water. The plate was left to dry for overnight; then, 2.5 ml of absolute alcohol 99.9% was added to the wells of the plate. From each well, 200 *µ*l was transferred to a 96-well plate, and optical density readings of the plate were measured at 590 nm using a Tecan Genios Pro microplate reader (Tecan Group Ltd, Männedorf, Austria).

#### 2.10.2. Effect of Extracts and Tormentic Acid Congener on Biofilm Detachment

Precultured *S. pyogenes* and *A. baumannii* cells were centrifuged at 3500 rpm for 15 minutes. The supernatant of the centrifuged cell culture was discarded, and 20 ml of 0.5 M phosphate-buffered saline prepared from sodium dihydrogen phosphate as the acidic salt and disodium hydrogen phosphate as the basic salt at pH 7.20 was used to wash the cells. The cell suspension was centrifuged again at 3500 rpm for 15 minutes, the supernatant was discarded, and 5 ml of sterile media was added and hand shaken so as the dissolve the cell pellet in the media. A 2 ml volume of standardised cells were added to each well of a 24-well plate (Sigma-Aldrich Co. Darmstadt, Germany). The cells were allowed to adhere to the wells of a 24-well microplate by incubating the cells in a shaking incubator at 37°C for 2 hours. After 2 hours of incubation, the microplate is incubated in a nonshaking incubator for 72 hours. After incubation, 1.2 ml of 100 *µ*g/ml of antibacterial test solutions and 1.2 ml of Tween 20 detergent solution were added to the plates. The plate was incubated in a nonshaking incubator, at 37°C for 2 hours; thereafter, the biofilm was quantified.

#### 2.10.3. Effect of Extracts and Tormentic Acid Congener on Capsular Polysaccharide Content of Biofilms

The extracellular polysaccharide of the extra polymeric substance was quantified using the phenol-sulphuric acid method. For the capsular polysaccharide analysis, precultured *S. pyogenes* and *P. aeruginosa* cells were standardised and added as 4 ml volumes to 5 sterilised 50 ml centrifuge tubes with 100 *µ*l of 100 *µ*g/ml of *C. viminalis* extracts and tormentic acid congener as well as 0.64 *µ*g/ml of the positive control, ciprofloxacin. The cells were further incubated in a shaking incubator at 37°C for 4 hours. After the incubation period, cells were separated by centrifugation at 4000 rpm for 15 minutes. The procedure was repeated thrice with chilled phosphate-buffered saline of pH 7.2. After centrifugation, the cells were suspended in 450 *µ*l of autoclaved distilled water, and an equal volume of 450 *µ*l of saturated phenol was added and then heated in a waterbath at 65°C for 20 minutes. For each sample, 300 *µ*l was taken in triplicate and added to sterile Eppendorf tubes. To each tube, 150 *µ*l chloroform was added, and the solution was mixed intensely by vortexing. The cell suspension mixture was then centrifuged, and 50 *µ*l of the supernatant was collected and distributed into their respective wells of a flat-bottomed microplate. A standard curve, using 50 *µ*l mannose at concentrations from 100 *µ*g/ml to 6.30 *µ*g/ml as well as a control with no mannose, was prepared. In each well, 150 *µ*l of concentrated sulphuric acid and 30 *µ*l of 5% phenol were also added, and the microtitre plate was put in a static waterbath of 90°C for 5 minutes. After heating, the plate was allowed to cool at room temperature for a further 5 minutes and wiped dry; absorbance was read at 492 nm using a Stat Fax 2 100 microplate reader (Awareness Technologies Inc, Westport, United States of America).

#### 2.10.4. Effects of Tormentic acid Congener on Extracellular DNA Content in Static Biofilms

The effect of tormentic acid congener, DCM:MeOH extract, and EtOH:H_2_O extracts on extracellular DNA production in static biofilm formation was studied using a method developed by Hawser and Douglas [33]. Briefly, precultured *P. aeruginosa* cells were centrifuged at 3500 rpm for 15 minutes. The pellet was washed using 0.5 M phosphate-buffered saline pH 7.2. The tormentic acid congener, DCM:MeOH extract, and EtOH:H_2_O extract were dissolved in 1% DMSO and Luria broth to give a final concentration of 12.5 *µ*g/ml. Of the resulting solution, 500 *µ*l was taken and added to the wells of a 24-well microplate. Positive control was set up using ciprofloxacin at a concentration of 0.80 *µ*g/ml. Negative control of media and cells was also run in parallel.

## 3. Results and Discussion

### 3.1. Isolation and of Identification of Tormentic Acid Congener

The following spectral characteristics were obtained: white powder; ^1^H-NMR (DMSO, 400 MHz) *δ* (ppm): 5.14 (1H, *br s*, H-12), 3.49 (1H, m), 2.74 (1H, *d*, *J* = 9.2, H-3), 2.12 (1H, *d*, *J* = 11.2, H-18), 2.00–1.20 (CH_2_ and CH region), 1.04 (3H, *s*, H-27), 0.93 (3H, *s*, H-23), 0.93 (3H, *s*, H-25), 0.92 (3H, *s*, H-30), 0.83 (3H, *d*, *J* = 6.4, H-29), 0.77 (3H, *d*, H-5), 0.75 (3H, *s*, H-26), and 0.72 (3H, *s*, H-24). ^13^C NMR (DMSO, 100 MHz) *δ* (ppm): 178.7 (C-28), 138.7 (C-13), 124.9 (C-12), 82.7 (C-3), 70.2 (C-20), 67.6 (C-2), 55.2 (C-5), 52.8 (C-18), 47.5 (C-17), 47.5 (C-1), 47.4 (C-9), 46.4 (C-14), 40.5 (C-10), 39.4 (C-8), 39.3 (C-4), 38.9 (C-21), 38.8 (C-19), 36.8 (C-22), 33.0 (C-7), 30.6 (C-16), 29.3 (C-23), 27.9 (C-15), 23.7 (C-27), 23.4 (C-11), 21.5 (C-30), 18.6 (C-6), 17.6 (C-24), 17.5 (C-26), 17.4 (C-24), 16.9 (C-29), and 16.9 (C-25).

The principle active showed the molecular ion (M^+^) at m/z 488, which agrees with the molecular formula C_30_H_48_O_5_. The ^1^H-NMR spectrum showed the presence of six singlet methyls and two doublet methyls, which were characteristic of the ursene skeleton, and exhibited signals of an olefinic proton (*δ* 5.14). The spectrum also showed a singlet at *δ* 3.49 (*m*) and two oxygen-bearing methine protons suggestive of the 2*α*, 3*β*, 19*α*-trihydroxy structure. These results indicated that the compound was an ursane-type triterpene. Furthermore, ^13^C NMR data substantiated the presence of a pair of olefinic carbons (*δ* 138.7 (C-13) and *δ* 124.9 (C-12)), a carboxylic acid group (*δ* 178.7 (C-28)), and three hydroxylated carbons (*δ* 82.7 (C-3), 70.2 (C-20), and 67.6 (C-2)) on the ursene structure. The structure was determined to be 2*α*, 3*β*, 20 *β*-trihydroxyurs-12-en-28-oic acid [34].

### 3.2. Antibacterial Susceptibility Tests

**Figure 1:**
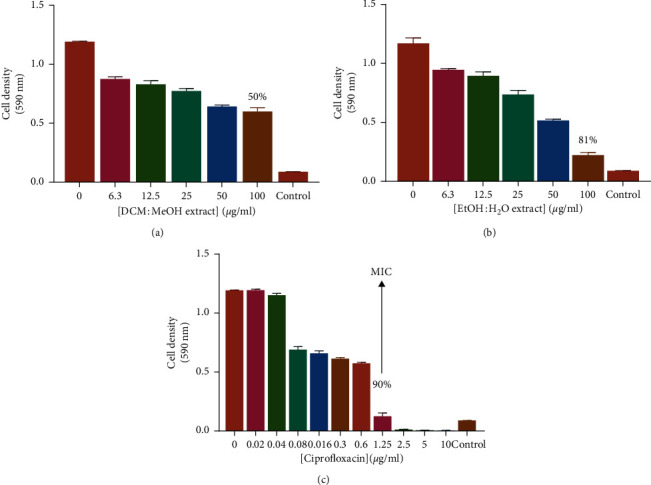
The effects of exposing on *K. pneumoniae* to extracts from *Callistemon viminalis*. Results of the microbroth dilution assay results are shown for (a) DCM:MeOH extract, (b) EtOH:H_2_O extract, and (c) ciprofloxacin. The error bars represent standard deviation from the mean of 4 repeat measurements.

The antibacterial effect of *C. viminalis* leaves was first screened by testing the effects of methanolic and ethanolic crude extracts on all five bacteria using the microbroth dilution assay. After incubation with MTT, the number of viable cells was determined spectrophotometrically. The antibacterial susceptibility tests showed *S. aureus* and *K. pneumoniae* (Figure 1**)** to be more susceptible to both extracts of *C. viminalis* than *P. aeruginosa*. These results are consistent with those of other studies which suggest that Gram-negative bacteria are less susceptible to xenobiotics than Gram-positive bacteria [35]. The DCM:MeOH extract and EtOH:H_2_O extract of *C. viminalis* may, however, have a broader spectrum of antibacterial activity as they exhibited activity against both Gram-positive and Gram-negative bacterial species.

Another important finding was that all extracts of *C. viminalis* had a minimal antibacterial effect against *P. aeruginosa* matching those observed in an earlier study by Chitemerere and Mukanganyama [36]. *P. aeruginosa* has a thick outer membrane that is highly hydrophobic, and this membrane provides a permeability barrier to the extract. The determination of MIC using *C. viminalis* crude extracts was an indicator of the effect of various triterpenes, flavonoids, and alkaloids that have antibacterial activity which are present in the plant leaves. At the highest concentration of tormentic acid congener (100 *µ*g/ml), there was complete inhibition of the bacterial activity of *P. aeruginosa*.

Extracts of *C. viminalis* were also tested for antibacterial activity against *S. pyogenes* and *A. baumannii*. The 50% v/v EtOH:H_2_O extract of *C. viminalis* and tormentic acid congener proved were shown to have the most potent antibacterial effects against *S. pyogenes* at 100 *µ*g/ml. Extracts of *C. viminalis* and tormentic acid congener did not have much inhibitory effects against *A. baumannii*, indicating intrinsic resistance of this bacterial strain. Antimicrobial resistance of *A. baumannii* is mainly due to reduced permeability of the outer membrane caused by loss or low porin expression, increased expression of multidrug efflux pumps, and mutations altering targets or different cellular functions [37]. The antibacterial effects of *β*-naphthoflavone on *A. baumannii* were also tested and were shown to have an inhibitory effect of 68%. Flavonoids are formed as antimicrobial barriers in plants response to microbial infection [38]. Flavonoids target the bacterial cytoplasmic membranes causing membrane fusion between microorganisms, resulting in leakage of intramembranous materials which promotes aggregation. Also, large bacterial aggregate clumps are more easily detected by the innate immune system compared to those bacteria in biofilm [39]. The poor activity of cell membrane active flavones against Gram-positive *S. pyogenes* might be because of poor penetration to the cell membrane of the bacteria due to presence of a thick layer of peptidoglycan in their cell walls which act as a barrier. Tiwari and colleagues showed that herbal compounds are generally antibacterial agents but show better antimicrobial activity when used in synergy with other antibiotics [37].

### 3.3. Time-Kill Kinetics of Tormentic Acid Congener on *P. aeruginosa*

**Figure 2:**
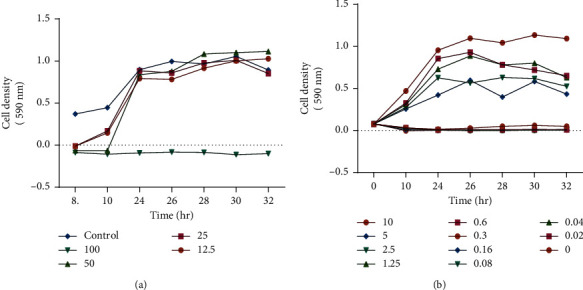
Time-kill curves of *P aeruginosa* cells after exposure to tormentic acid congener (a) and ciprofloxacin (b). Cells were standardised to 1 × 106 cfu/ml. They were incubated with the compounds at 2-fold increasing concentrations for 32 hours and incubated at 37°C.

The time-kill assay was carried out to determine the capacity of tormentic acid congener to kill the bacterium *P. aeruginosa* in relation to time. A range of five concentrations (100–0 *µ*g/ml) were used, together with ciprofloxacin as a control. The antibacterial effect of tormentic acid congener on *P. aeruginosa* was shown to be time-dependent. At the highest concentration of tormentic acid congener 100 *µ*g/ml, there was a complete inhibition of the bacterial activity of *P. aeruginosa* (Figure 2(a)). At 100 *µ*g/ml, tormentic acid congener completely inhibited the growth of *P. aeruginosa* cells. The antibacterial activity of tormentic acid congener was efficient with 50 *µ*g/ml inhibiting the growth of *P. aeruginosa* during the first 10 hours. The positive control ciprofloxacin showed an inhibition of the growth of *P. aeruginosa* cells from a minimum concentration of 0.32 *µ*g/ml–10 *µ*g/ml. There was a subsequent increase in cell growth in the wells with 0.16, 0.08, 0.04, and 0.02 *µ*g/ml of tormentic acid congener, although the rate of cell growth was lower than that of the standard control. The time-kill assay of ciprofloxacin further confirmed the minimum inhibitory concentration of 0.32 *µ*g/ml against *P. aeruginosa* (Figure 2(b)).

### 3.4. Effects of Combining Tormentic Acid Congener and Ciprofloxacin on the Growth of *P. aeruginosa*

**Figure 3:**
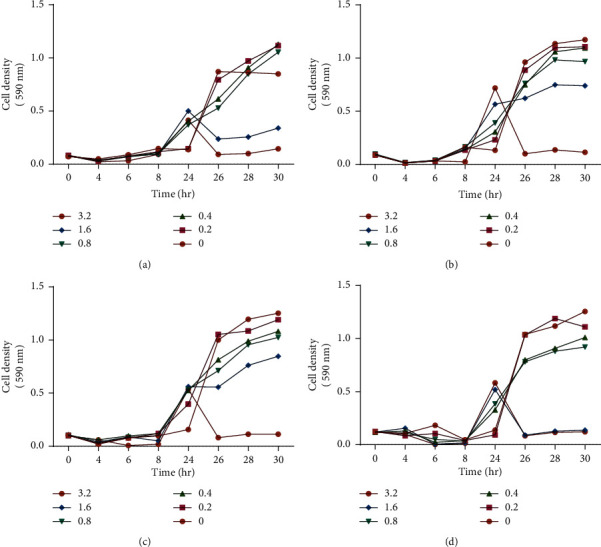
The effects of combining tormentic acid congener and ciprofloxacin on *P aeruginosa*. The effect of ciprofloxacin on *P aeruginosa* together with a concentration of (a) 0 *µ*g/ml, (b) 25 *µ*g/ml, (c) 50 *µ*g/ml, and (d) 100 *µ*g/ml of tormentic acid congener.

Antibacterial combinations have been extensively researched to determine the synergistic combination and interactions between potential antibacterial agents to optimise the efficacy and potency of antibacterial drugs [40]. Synergism occurs when there is a decrease in the viable organism as an outcome of combining two antibiotics when compared to the effect of using the most effective antibiotic alone [41]. The checkerboard combination assay was carried out to determine the effect of combining ciprofloxacin and tormentic acid congener. For tormentic acid congener, the MIC, ½ MIC, ¼ MIC, and ⅛ MIC was used. The concentrations of ciprofloxacin used were MIC 0.32, 0.16, 0.08, 0.04, and 0.02. As shown in Figure 3, tormentic acid congener decreased the MIC of ciprofloxacin from 0.3 *µ*g/ml to 0.16 *µ*g/ml at the MIC concentration of 100 *µ*g/ml. The other concentrations of tormentic acid congener did not have a significant effect on the MIC of ciprofloxacin.

Combining concentrations of 100 *µ*g/ml of tormentic acid congener and 0.016 *µ*g/ml of ciprofloxacin was indifferent at 37°C for 3 hours incubation because the calculated FICI was 0.5016. Some in vitro tests have indicated that terpenes show ineffective antimicrobial activity when used as a single compound but are more effective when used in combination with other antibacterial agents [42]. Another study showed synergism when triterpenes were combined with ciprofloxacin and tested on *E. coli* [43]. Several diterpenoids, terpenoids, and sesquiterpenoids have been found to act synergistically with different antibiotics [44]. Similarly, tormentic acid congener might be able to work in synergy with other antibiotics regardless of its inability to work in synergy with ciprofloxacin. Some of the antibacterial drugs that show synergistic or additive effects when combined with triterpenoids include methicillin and vancomycin [45].

### 3.5. Determination of the Effects of Tormentic Acid Congener on Membrane Integrity Using the Protein Leakage Determination Assay

**Figure 4:**
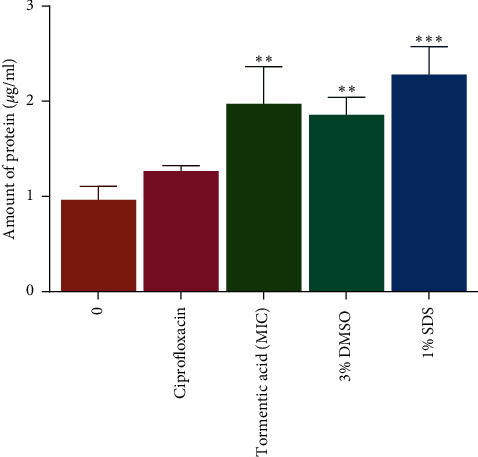
The effect of exposing *P aeruginosa* to ciprofloxacin (0.016 *µ*g/ml) and tormentic acid congener (100 *µ*g/ml) on release of intracellular protein in *P. aeruginosa*. The absorbance intensities of treated *P aeruginosa* cells were measured at 590 nm. Results are presented as mean (*n* = 3) ± standard deviations. Asterisk denotes a significant difference (^∗∗^*P* < 0.01).

The proposed mechanism of action of tormentic acid congener was protein leakage, and this was determined by exposing the bacteria to the compound and determining the amount of protein using the Bradford assay. The mechanism of action of ciprofloxacin against bacteria is not cell lysis, and therefore, ciprofloxacin did not cause significant cell lysis in *P. aeruginosa* in comparison to the unexposed cells. As shown in Figure 4, tormentic acid congener caused cell lysis in *P. aeruginosa*, and this exposure resulted in significant protein leakage. The positive controls were 1% SDS that caused significant protein leakage in *P. aeruginosa*. Exposure to ciprofloxacin did not result in protein leakage, and this was in correlation with the research that was performed by Jedrey et al. [46]. SDS caused notable cell lysis in *P. aeruginosa* cells [47], which resulted in protein leakage.

Studies have shown that some phytochemicals including terpenes affect the stability of the cell membrane in bacterial cells, and these include p-cymene, carvacrol, and thymol [43]. Carvacrol for instance causes functional and structural damage and disruption of the membrane Gram-negative pathogens [43]. Thymol merges to the polar headgroups that make up the lipid bilayers, and it induces alterations to the cell membrane affecting its permeability [43]. Other terpenes that have effectively damaged bacterial cells of *S. aureus* and *E. coli*, respectively, are citronellol and citronellal, and their permeabilisation to the membrane or cell wall are related to alterations on their physicochemical properties [48].

### 3.6. Effects of Extracts and Tormentic Acid Congener on Species on Biofilm Production

**Figure 5:**
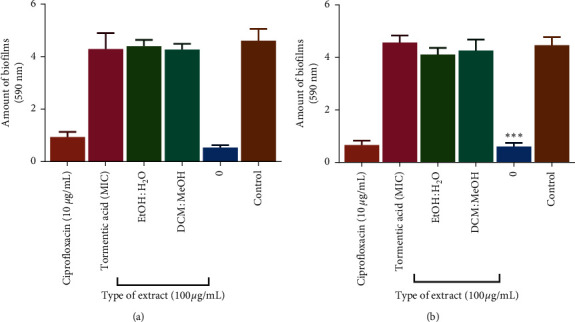
The effect of ciprofloxacin, tormentic acid congener, and *C. viminalis* extracts on *S pyogenes* (a) and *A. baumannii* (b) biofilm production, respectively. The bacterial strains were incubated together with the test agents for 72 hours, and the formed biofilm was quantified using 0.1% crystal violet. Ciprofloxacin inhibited the formation of biofilms in both bacteria, whilst tormentic acid congener and *C. viminalis* extracts showed no significant inhibitory effect. Asterisk denotes a significant difference (^∗∗∗∗^*P* < 0.0001).

Biofilm formation is a major resistance mechanism displayed by bacteria. Both *S. pyogenes* and *A. baumannii* can form biofilms [49, 50]. The extracellular polymeric substance is composed of polysaccharide, proteins, and extracellular DNA [51]. Biofilm formation activities were determined using 0.1% crystal violet staining of adherent biofilm, and the results are shown in Figure 5. To evaluate the effect of *C. viminalis* extracts, tormentic acid congener, and ciprofloxacin against biofilm formation, the antibiofilm agents were incubated together with the bacterial strains in a 24-well plate for 72 hours at 37°C. Ciprofloxacin inhibited biofilm formation by 80% and 85% in *S. pyogenes* and *A. baumannii*, respectively. Tormentic acid congener and *C. viminalis* extracts had no significant effect on biofilm formation inhibition for both test bacterial strains. Biofilms account for over 80% of microbial infections in the human body [52]. Bacterial cells in biofilms are 10–1000 times less susceptible to antimicrobial agents compared to their planktonic counterparts due to the physical impedance leading to poor diffusion of the drugs into the biofilm [53]. Also, bacteria embedded in a biofilm can evade the host immune system, therefore, contributing to its resistance mechanism [54]. Most bacteria exist within biofilms encased in an extracellular polymeric substance made up of biopolymers [55].

#### 3.6.1. Effects of Extracts and Tormentic Acid Congener Detachment of Preformed Biofilms

**Figure 6:**
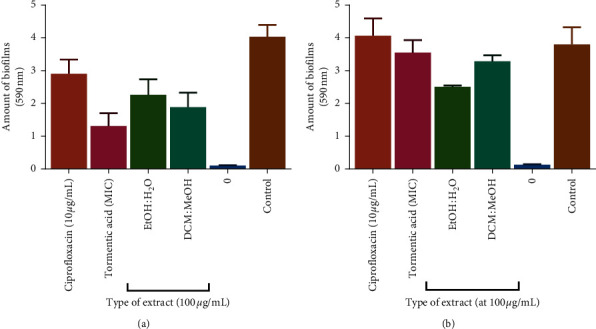
The effect of ciprofloxacin, tormentic acid congener, and *C. viminalis* extracts on biofilm detachment in *S. pyogenes* (a) and *A. baumannii* (b). Ciprofloxacin showed less effect on preformed biofilms than *C. viminalis* extracts and tormentic acid congener. Tormentic acid congener caused detachment of biofilms in *S. pyogenes* more than other agents, whilst the EtOH extract of *C. viminalis* had the greatest effect in *A. baumannii*.

The standard antibiotic drug ciprofloxacin showed the least antiadhesion properties on the already formed biofilms in both test bacteria, *S. pyogenes* and *A. baumannii* (Figure 6). Ciprofloxacin only caused the detachment of 28% of the biofilm formed in *S. pyogenes* and had no detachment effect on biofilms formed by *A. baumannii*. Tormentic acid congener and *C. viminalis* extracts had no biofilm formation inhibitory effect but rather cause detachment of already formed biofilms in both *S. pyogenes* and *A. baumannii*. Lack of inhibitory effect on biofilm formation could be attributed to the low antibacterial activity of the test antimicrobial agents. *A. baumannii* proliferated at 100 *µ*g/ml of test antibacterial agents and produced biofilms at same concentrations increasing its mechanisms of resistance. Ciprofloxacin showed antibiofilm activity at 10 *µ*g/ml of the drug. The subminimum inhibitory concentration of ciprofloxacin was supposed to be used to ensure bacterial species were not killed by high ciprofloxacin concentrations. Ciprofloxacin had no biofilm detachment activity against test bacterial species as shown by tormentic acid congener and *C. viminalis* extracts.

Cell surface hydrophobicity is a crucial factor for biofilm formation and stabilisation in biofilm-forming bacteria [56]. The hydrophobic property of bacterial surfaces is a major determinant in the adhesion of bacteria and the formation and stabilisation of biofilms by bacteria on animate and inanimate surfaces [56]. Nandu et al., established that fukugiside, a biflavonoid isolated from the leaves of *Garcinia travancorica*, reduced the cell surface hydrophobicity of *S. pyogenes* as a mechanism of its antibiofilm activity [55]. Therefore, tormentic acid congener could also be reducing the hydrophobicity of the cell surface of *S. pyogenes* thus causing the detachment of already formed biofilms. It has also been suggested by Lim and colleagues that the detachment of biofilms could be as a result of reduced adhesive forces in the biofilms due to increased solubility of bacterial exopolysaccharides [56]. Tormentic acid congener and 50% v/v EtOH:H_2_O extract of *C. viminalis* disrupted the biofilm architecture *S. pyogenes* and *A. baumannii*, respectively.

#### 3.6.2. Effect of Tormentic Acid Congener on Extracellular DNA Production in Biofilms

**Figure 7:**
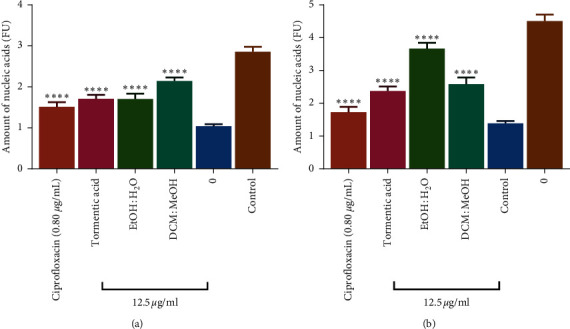
The effect of *C. viminalis* EtOH:H_2_O extract, DCM:MeOH extract, tormentic acid congener, and ciprofloxacin on extracellular DNA produced by *P. aeruginosa* (a) and *S. aureus* (b). Cells were incubated in a medium containing 0.80 *µ*g/ml ciprofloxacin as well as 12.5 *µ*g/ml of the crude extracts and tormentic acid congener for 72 h. The amount of extracellular DNA present was determined by staining the biofilms with propidium iodide and spectrophotometrically measuring the amount of dye. These are results after a 24 well plate was washed with phosphate-buffered saline. ANOVA was carried out using GraphPad Prism6 and Dunnett's multiple comparisons test with a summary of ^∗∗∗∗^*P* value < 0.0001. Ciprofloxacin, tormentic acid congener, and extracts reduced extracellular DNA production in *P. aeruginosa* and *S. aureus* biofilms.

Most bacteria exist within biofilms encased in an extracellular polymeric substance made up of biopolymers [57]. This present study investigated the antibacterial effects of tormentic acid congener and different *C. viminalis* crude extracts as well as the inhibition of extracellular polysaccharide production against *S. aureus* and *P. aeruginosa*. The results for the effects of the extracts and tormentic acid congener on the production of extracellular DNA from *P. aeruginosa* (Figure 7(a)) and S*. aureus* (Figure 7(b)) are shown. Ciprofloxacin, extracts, and tormentic acid congener reduced extracellular DNA production in the biofilms. As shown in Figure 7(a), ciprofloxacin reduced significantly more the extracellular DNA produced by *P. aeruginosa* compared to tormentic acid congener and the crude extracts. Tormentic acid congener does, however, significantly reduced extracellular DNA more than the crude extracts. The EtOH:H_2_O extract was more potent than the DCM:MeOH extract in reducing extracellular DNA in *P. aeruginosa* biofilms. Data in Figure 7(b) show that ciprofloxacin and tormentic acid congener had similar effects on extracellular DNA formed by *S. aureus* biofilms as those shown with *P. aeruginosa*. The DCM:MeOH was, however, more potent than the EtOH:H_2_O extract in reducing extracellular DNA in *S. aureus* biofilms.

Extracellular DNA (eDNA) was observed to be abundant in the biofilm matrix as shown in a study by Flemming et al. [58]. A study by Tang et al. showed that eDNA had many functions such as the initial attachment of biofilms of *P. aeruginosa*, *Reinheimera*, *Microbacterium,* and *Serratia* species [59]. Due to the complex structure of the biofilm, eDNA may provide inherent resistance to antimicrobial agents. The search for an effective drug to eradicate the biofilms or inhibit their growth by targeting the inhibition of eDNA is of importance. Our results showed that the amounts of extracellular DNA produced by *S. aureus* and *P. aeruginosa* strains were significantly inhibited by tormentic acid congener and *C. viminalis* crude extracts.

#### 3.6.3. Effects of Tormentic Acid Congener on Capsular Polysaccharide Production

**Figure 8:**
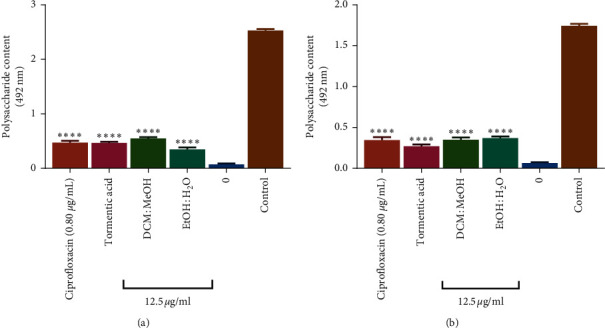
The effect of *C. viminalis* EtOH:H_2_O extract, DCM:MeOH extract, tormentic acid congener, and ciprofloxacin on biofilm polysaccharide content. *P. aeruginosa* (a) and *S. aureus* (b) were incubated in a medium containing 0.80 *µ*g/ml ciprofloxacin and 12.5 *µ*g/ml of extracts and tormentic acid congener for 16 hours. Mannose was used as the standard for carbohydrate content. Carbohydrate content was read at 492 nm using a Stat Fax model microplate reader. ANOVA was carried out using GraphPad Prism6 and Dunnett's multiple comparisons test with a summary of ^∗∗∗∗^*P* value < 0.0001.

**Figure 9:**
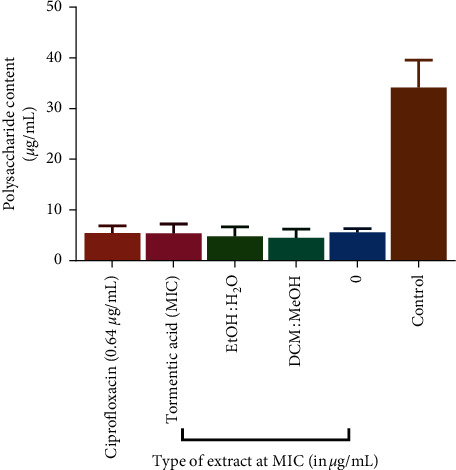
The effect of ciprofloxacin, tormentic acid congener, and *C. viminalis* extracts on extracellular polysaccharide content in *S. pyogenes*. Quantification of extracellular polysaccharide content of *S. pyogenes* was performed using the 5% phenol saturated sulphuring acid test and collection and measuring of the supernatant. All test agents inhibited the formation of capsular exopolysaccharides in *S. pyogenes*.

Polysaccharides act as molecular glue in biofilm formation so the reduction in polysaccharide production implies a reduction in biofilm formation and thus provides a possible alternative to killing or inhibition of the growth of pathogenic bacteria [60]. In order to determine if tormentic acid congener, ciprofloxacin, DCM:MeOH extract, and EtOH:H_2_O extracts had any effect on the formation of biofilms in *P. aeruginosa* and *S. aureus*, the capsular polysaccharide production was quantified after exposure to these samples. The EtOH:H_2_O extract showed the most significant reduction in polysaccharide formation when compared to the reductions caused by tormentic acid congener and DCM:MeOH extract (Figure 8). The EtOH:H_2_O extract was more potent than the DCM:MeOH extract in reducing polysaccharide production in *P. aeruginosa* biofilms (Figure 8(a)). The DCM:MeOH showed more potency in reducing polysaccharide production than the EtOH:H_2_O extract in *S. aureus* (Figure 8(b)). All antibacterial test agents reduced the extracellular polysaccharide content in *S. pyogenes* biofilms (Figure 9).

Bacteria in biofilms have been shown to produce extracellular polysaccharides (EPS) which help them to bind the biofilm together in a matrix while anchoring it to surfaces [61]. For this study, it is interesting to note that EPS production is significantly reduced for both Gram-positive and Gram-negative pathogens in the presence of tormentic acid congener and different *C. viminalis* crude extracts. This presents a result that has some importance for the possible use of tormentic acid congener as a biofilm control agent. The regulation of EPS production has been studied well for both Gram-positive and Gram-negative pathogens. In a study by Olofsson et al. [62], it was shown that Gram-negative bacteria extracellular polysaccharide production is inhibited directly or indirectly due to naturally occurring antibacterial agents. Similar findings of reduction of EPS against Gram-positive pathogens were reported for the methanol leaf extract fraction of *Mangifera indica* [63]. Our results also demonstrate a reduction in EPS production after exposure to plant extract, thus resulting in inhibition of biofilm production. This study showed that exposing in *S. aureus* and *P. aeruginosa* to tormentic acid congener and extracts from C*. viminalis* results in decreases in EPS and eDNA production which may result in decrease in biofilm formation.

## 4. Conclusion

*C. viminalis* extracts had antibacterial activity against *P. aeruginosa*, *S. aureus*, *S. pyogenes*, *K. pneumoniae*, and *A. baumannii*. Extracts of *C. viminalis* reduced polysaccharide production and extracellular DNA production in *P. aeruginosa* and *S. aureus* biofilms. Tormentic acid congener caused significant cell lysis in *P. aeruginosa* cells. Exposure to tormentic acid congener produced detachment of *S. pyogenes* biofilms well as it reduced the extracellular polysaccharide content of *S. pyogenes* biofilms. However, tormentic acid congener and both *C. viminalis* extracts did not show significant antibacterial activity and antibiofilm formation activity against *A. baumannii.* Tormentic acid congener and extracts of *C. viminalis*, thus, had significant antibacterial and antibiofilm activities on selected ESKAPE bacteria and can act as source lead compounds for the development of antibacterial triterpenoids.

## Data Availability

The datasets used and/or analysed during the current study are available from the corresponding author upon request.
